# Multilevel analysis of factors associated with untreated diarrhea among under five children in Ethiopia using Ethiopian demographic and health survey

**DOI:** 10.1038/s41598-023-43107-9

**Published:** 2023-09-26

**Authors:** Tewodros Getaneh Alemu, Elsa Awoke Fentie, Desale Bihonegn Asmamaw, Ever Siyoum Shewarega, Wubshet Debebe Negash, Habitu Birhan Eshetu, Daniel Gashaneh Belay, Fantu Mamo Aragaw, Samrawit Mihret Fetene, Rediet Eristu Teklu

**Affiliations:** 1 Department of Pediatrics and Child Health Nursing, School of Nursing, College of Medicine and Health Sciences, University of Gondar, Gondar, Ethiopia; 2 Department of Health Systems and Policy, Institute of Public Health, College of Medicine and Health Sciences, University of Gondar, Gondar, Ethiopia; 3 Department of Epidemiology and Biostatistics, Institute of Public Health, College of Medicine and Health Sciences, University of Gondar, Gondar, Ethiopia; 4 Department of Reproductive Health, Institute of Public Health, College of Medicine and Health Sciences, University of Gondar, Gondar, Ethiopia; 5 Department of Health Promotion and Health Behavior, Institute of Public Health, College of Medicine and Health Sciences, University of Gondar, Gondar, Ethiopia; 6 Department of Human Anatomy, College of Medicine and Health Sciences, University of Gondar, Gondar, Ethiopia

**Keywords:** Diseases, Health care, Risk factors

## Abstract

Diarrhea refers to the abrupt onset of three or more loose or liquid stools per day. It is the second leading cause of death in infants worldwide. It is an endemic disease and continues to be a serious threat to children in Ethiopia. Despite being a condition that may be prevented, diarrhea can have a negative impact on a child's health. Also, studies have not been able to explore the role of socio-economic characteristics in hindering the treatment. Therefore, this study aimed to explore socio-economic factors that influence treatment of childhood diarrhea. Secondary data analysis was conducted based on the demographic and health surveys data conducted in Ethiopia. A total weighted sample of 1227 under-five children was included for this study. Mixed-effect binary logistic regression analysis was done to identify associated factors of untreated diarrhea. Adjusted Odds Ratio with 95% CI was used to declare the strength and significance of the association. Prevalence of untreated diarrhea among under five children in Ethiopia was 57.32% (95% CI 54.52–60.06%). In the mixed-effect analysis; Children aged 6–11, 12–23, and 24–35 (AOR 0.384, 95% CI 0.187–0.789), 71% (AOR 0.29, 95% CI 0.149–0.596), and 51% (AOR 0.49, 95% CI 0.238–0.995). Children from family number six and above (AOR 1.635, 95% CI 1.102–2.426). Children from middle wealth of family (AOR 1.886, 95% CI 1.170–3.3040). Children from a community with high level of uneducated (AOR 2.78, 95% CI 1.065–3.442) were significantly associated with untreated diarrhea. The prevalence of untreated diarrhea among under-five children in Ethiopia is high. Age of child, family number, household wealth, and community-level educational status were significantly associated with untreated diarrhea among under-five children in Ethiopia. Hence, increasing community educational status, boosting the economic status of the community, and family planning for the community should get due attention.

## Introduction

Children are the most vulnerable age group in any community; hence, the under-five mortality rate is a widely used demographic measure and an important indicator of the level of welfare in countries^1^. However, Diarrhea is the second leading cause of death in infants and kills approximately 525,000 toddlers annually worldwide^2,3^. Globally, there are nearly 1.7 billion cases of diarrhea in toddlers every year^3^. Despite this heavy toll, progress is being made. From 2000 to 2015, the total annual number of deaths from diarrhea among children under 5 decreased by more than 50 percent from over 1.2 million to half a million and by 60% in Ethiopia between 2000 and 2016^4,5^. In the face of these gains, however, Diarrhea is an endemic disease in Ethiopia; revealed that the pooled prevalence of diarrhea among under-five children in Ethiopia was 22%^6^. Diarrhea refers to the abrupt onset of three or more loose or liquid stools per day^7^. To be more precise, acute diarrhea is described as an abnormally frequent, short-term (less than 14 days), semi-solid or fluid fecal matter discharge from the intestine^8^. For the treatment of diarrheal disease, a variety of interventions are available, including as oral rehydration solutions, zinc treatment, continuous feeding, and antibiotic treatment for particular strains of diarrhea^9,10^. Oral rehydration salts (ORS) and, more recently, Zinc tablets have been shown to be more cost-efficient and effective as primary interventions for preventing diarrhea morbidity^11,12^. Despite the availability of these interventions; today, globally only about 40% of children receive adequate healthcare^13^ when diarrhea symptoms arise^14^.

In sub-Saharan Africa, only about one in three children experiencing diarrheal episodes receives ORS, and the proportion receiving zinc is below 5%^15^. Furthermore, many children are not using the interventions in Ethiopia^16^. As a result, diarrhea disease continues to be a serious threat to children in Ethiopia^6^. Diarrhea, despite being a preventable condition, can have serious health consequences for children. Diarrhea, for example, causes dehydration, which can lead to malnutrition, development impairment, or mortality in children under the age of five, who are particularly prone to disease^17^. Furthermore, research shows that children who have diarrhea before their second year are 5% more likely to be stunted than their peers who do not^18^. Aside from these, diarrhea diseases have been linked to long-term negative mental consequences as well as decreased economic or work efficiency in later life^19,20^.

Health care-seeking behavior and the choices for treatment are influenced not only by the traditional beliefs but also by socio-demographic factors^21^. However, the relative importance of the influence of social or economic position on health-seeking behavior and treatment varies among countries with different socio-demographic and economic contexts^22–24^. No formal education, poor socio-economic status, mothers who have no work were known contributing factors for untreated diarrhea in children^24,25^. In 2015, the United Nation adopted the Sustainable Development Goals^26^ to reduce child mortality and to promote well-being for all children. The SDG goal #3 Target 3.2 aims to end preventable deaths of newborns and under-five children by 2030^26^. Likewise, the Ethiopian government also implemented various strategies, such as the Health Extension Program, and integrated community case management (ICCM) to prevent and control infectious diseases like diarrhea^27^. Despite interventions and innovations by a range of stakeholders, under-five mortality related to diarrhea remains a major concern, especially in developing countries like Ethiopia^6^.

Although several studies have documented the role of socio-economic factors in care-seeking for childhood diarrhea^28,29^. Many of these studies have not been able to explore the role of socio-economic characteristics in hindering the treatment^12,30^. Furthermore, to the best of our knowledge, there has been no research to date that has rigorously examined the association between socio-economic factors and untreated diarrhea among under five children in Ethiopia. Additionally, there has not been newly implemented program since 2016 that improved diarrhea related problems in the country. Thus, this study aimed to explore socio-economic factors that influence on treatment of childhood diarrhea in Ethiopia. Therefore, detecting factors that influence untreated diarrhea during this age is important to prioritize and design targeted intervention programs to reduce the number of untreated diarrhea and diarrhea associated mortality in children.

## Methods

### Study design and setting

A population-based repeated cross-sectional study was used to investigate the socio-economic determinants of untreated childhood diarrhea. This is based on data from Ethiopian Demographic Health Survey (EDHSs) surveys that were conducted in 2016. The Demographic Health Survey (DHS) is a multi-round cross-country survey that evaluates population health with a focus on maternal and child health, as well as population health indicators of global importance. The data was gathered in collaboration with Ethiopia's Central Statistical Agency^5^ and Ministry of Health. Ethiopia is a country located in the horn of Africa. The total land area is estimated to be 1,126,829 km^2^. As a landlocked country, it is bordered by Djibouti, Eritrea, Kenya, Somalia, South Sudan, Sudan, and Somaliland (Somalia). Each of the country’s nine administrative regions and two administrative cities is organized into zones, districts, towns, and kebeles (the smallest administrative units).

### Data sources, sampling technique, and study population

The data for this study were drawn from recent nationally representative DHS data conducted in Ethiopia. A stratified two-stage cluster sampling technique was applied; with clusters being chosen first from 645 EAs (enumeration areas) and households being picked second. With clusters, enumeration areas sample households were chosen, and cluster selection was stratified by place of residence (rural/urban) and districts. In this study, all living under-five children living with their mother during the survey period were included and all living under-five children living with their mother was the study population in the selected enumeration areas. A child who is not alive and a child without diarrhea preceding the survey were excluded. The total weighted sample size from data analyzed in this study was 1227.

### Study variable

#### Dependent variables

Treatment of diarrhea: children with diarrhea received treatment within two weeks period prior to the survey was declared he or she received the treatment and recoded as “0” whereas no received treatment within two weeks period prior to the survey was declared he or she not received the treatment and recoded as “1”.

#### Independent variables

The independent variables were identified from various literature and these cover factors related to a child, maternal, household, and community characteristics that impact children’s treatment for diarrhea^17,22,24,25^. The child characteristics included age in a month (0–6, 6–11, 12–23, 24–35, > 35) and twin child (yes and no). The household and maternal characteristics included under five number of children in the household (2 or less, and 3 and above), mother occupation (not working and working), wealth index (poor, middle, and rich), marital status (not married and married), and age of the women in a year (15–24, 25–34, and 35–49), number of family (≤ 5, and 6 and above), a child lives with whom (mother and lives elsewhere), and religions (Catholic, Orthodox, Protestant, Muslim, and Tradition), health insurance of the family (no and yes), mothers education level (no formal education and formal education) and media exposure (media exposure was created from the four variables: watching television, listening radio, reading a newspaper, and use of internet and labeled as yes if a woman has exposure to either of the four media sources or no if a woman has exposure to none of them).

Community-level variables: In this study, place of residence, region (categorized as metropolitan, small peripheral, and large peripheral), regions that included metropolitan were Harari, Addis Abeba, and Dire Dewa, regions included in small peripheral were Afar, Somalia, Benishangul, and Gambela, and regions included in large peripheral were Tigray, Amhara, Oromia, and South nation and nationality. Distance of health facility (as “big problem” or “not a problem”), a big problem indicates that the distance from women’s home to health to get medical help for herself was problematic. If the women said that the distance was a big problem, the code was 0; if they said that it was not a big problem, the code was 1. While community-level women’s education, community-level media exposure, and community poverty level were constructed through the aggregation of individual-level factors to conceptualize their neighborhood effect on the treatment of diarrhea. The aggregated community level predictor variables were constructed by aggregating individual level values at cluster level, and binary categorization (high or low) of the aggregated variables were done based on the distribution of the proportion values calculated for each cluster. Median for not normally distributed community-level aggregated predictor variables were used as cut-off point for categorization. A histogram was used to check the distribution whether it is normal or not.

### Data collection tool, procedure, and data quality control

This study was a secondary data analysis based on datasets from the most recent DHS conducted in Ethiopia. The DHS is a nationally representative survey that collects data on basic health indicators like fertility, reproductive health, maternal and child health, mortality, morbidity, nutrition, and self-reported health behaviors among adults. This study used the recent DHS data done in Ethiopia. Used Kids Record (KR) files, which contain information about women and children, for this specific research, and extracting important variables related to diarrhea treatment were performed from the data set.

### Data management and analysis

The data were extracted, recoded, and analyzed using Stata 14 software^31^. Weighting was done to the survey data and create descriptive and summary statistics. For continuous explanatory variables that defy normality, median and interquartile range were chosen. Cross-tabulation was also carried out to display the percentage of various categories of each attribute in relation to the outcome variable. The typical assumptions of the logistic regression model might be violated because the DHS data is hierarchical and children ages 0–59 months were nested within-cluster and to restore the survey’s representativeness the sample weights were applied to compensate for the unequal probability of selection between the clusters and the outcome variable is binary. Therefore, we employed a multilevel logistic regression analysis. At the individual and community levels, multivariable multilevel logistic regression was performed to investigate factors associated with untreated diarrhea in children. In the analysis, four models were used. The null model, which does not include any explanatory factors, assesses the degree of the cluster variation on untreated diarrhea in children. The first model contains only individual-level variables, the second model contains only community-level variables, and the third model contains both individual-level and community-level variables. A P-value of < 0.05 was used to define statistical significance. Adjusted Odds Ratios (AOR) with their corresponding 95% confidence intervals^32^ were calculated to identify the independent predictors of untreated diarrhea in children. To evaluate the variation between clusters, the intra cluster correlation coefficient (ICC), Median Odds Ratio (MOR), and proportional change in variance (PCV) statistics were calculated. MOR is a measure of unexplained cluster heterogeneity, while ICC was employed to explain cluster variation. PCV measures the total variation attributed by individual-level factors and community-level factors in the multilevel model as compared to the null model PCV. For Model comparison log-likelihood and deviance were used.

### Patient and public involvement statement

Patients (participants) and the general public were not consulted during the study's conception or preparation. Patients were also not consulted to interpret the results because we used secondary data (DHS), and they were not invited to contribute to the creation or editing of this publication for readability or correctness.

### Ethical approval and consent to participate

Ethics approval was not required for this study since the data is secondary and is available in the public domain. To conduct our study, we registered and requested the dataset from DHS online archive and received approval to access and download the data files. This study was done in accordance with the relevant guidelines and regulations.

## Results

### Socio-demographic and economic characteristics of participants

From the DHS dataset, a total of 1227 children were included in this study. Half (52.87%) of the children were males. Nearly one-third of the children were between the ages of 12–23 months. The median age of the children was 14 (IQR 10, 18) months. Most of the mothers (93.97%) were married. Approximately 63% of the mothers had no formal education. Around 43.81% of the children born from poor household wealth status (Table 1).

**Table 1:** Socio demographic and economic characteristics of under five children and their caregiver in Ethiopia (n = 1227).

Variables	Categories	Weighted frequency	Percentage
Age of child	0–6 m	91	7.47
	6–11 m	241	19.60
	12–23	357	29.08
	24–35 m	250	20.38
	> 35	288	23.47
Sex of the child	Male	649	52.87
	Female	578	47.13
Child is twin	No	1195	97.40
	Yes	32	2.60
Child lives with whom	Mother	1213	98.82
	Lives else where	14	1.18
Mother occupation	Not working	648	52.82
	Working	579	47.18
Marital status of mother	Not married	74	6.03
	Married	1153	93.97
Sex of the household head	Male	1085	88.43
	Female	142	11.57
Under-five children number	2 or less	1075	87.66
	3 and above	152	12.34
Family size	≤ 5	610	49.72
	6 and above	617	50.28
Mother education	No formal education	767	62.46
	Formal education	460	37.54
Media exposure	Yes	426	34.73
	No	801	65.27
Wealth index	Poor	538	43.81
	Middle	267	21.79
	Rich	422	34.40
Age of women	15–24	307	24.97
	25–34	671	54.70
	35–49	249	20.32
Mother religion	Catholic	10	0.78
	Orthodox	451	36.71
	Protestant	313	25.57
	Muslim	429	34.95
	Tradition	24	1.99
Health insurance	No	1225	99.84
	Yes	2	0.16

### Community-level variables in Ethiopia DHS

More than three-fourth of the children (89.73%) were rural dwellers. Nearly half of the community (49.09%) were grouped in the higher community-level poverty. The highest number of children (93.47%) were from large central region. More than half of the children (58.28) distance to a health facility was a big problem (Table 2).

**Table 2:** Community-level variables in Ethiopia DHS (n = 1227).

Variables	Categories	Weighted frequency	Percentage
Community level poverty	Higher	603	49.09
	Lower	624	50.91
Community level education	Higher	604	49.16
	Lower	623	50.84
Region	Metropolitan	26	2.08
	Small peripheral	54	4.45
	Large central	1147	93.47
Residence	Urban	126	10.27
	Rural	1101	89.73
Community level media	Lower	674	54.93
	Higher	553	45.07
Distance to health facility	Not big problem	512	41.72
	Big problem	715	58.28

### Prevalence of untreated diarrhea among under-five children

**Figure 1:**
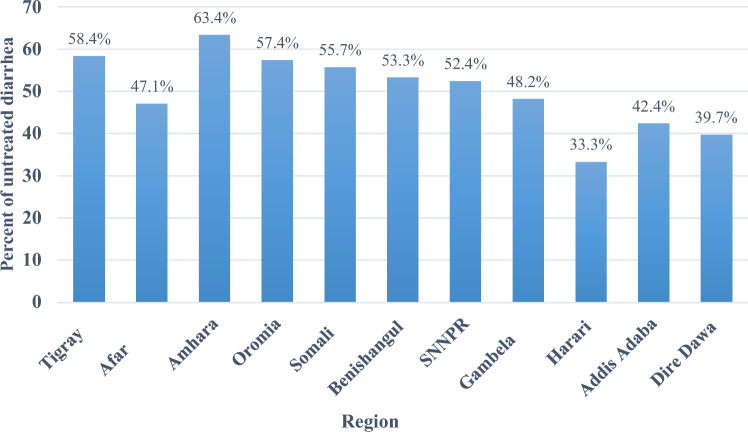
Regional prevalence of untreated diarrhea in Ethiopia.

Overall, 57.32% (95% CI 54.52–60.06%) of under-five children had untreated diarrhea in Ethiopia. Regionally, the prevalence of untreated diarrhea was highest in the Amhara region (63.40%), while it was lowest in the Harari region (33.30%) (Fig. 1).

### Random effect analysis and model comparison

The ICC value in the null model indicates 40.17% of the total diarrhea treatment. This was due to the difference between clusters. Besides, the high MOR value in the null model was 4.21. This revealed that when we randomly select children from two clusters, children’s from a high-risk cluster were 4.21 times more likely not to received treatment for diarrhea as compared to children’s from a low-risk cluster. Moreover, the PCV in the final model revealed that about 20% of the variability in untreated diarrhea was explained both by individual and community level factors. Regarding model fitness, model III was the best-fit model since it had the lowest deviance (Table 3).

**Table 3:** Random effect model and model fitness for the assessment of untreated diarrhea among under-five children in Ethiopia.

Parameter	Null model	Model 1	Model 2	Model 3
ICC	40.17	37.90	39.79	34.92
MOR	4.21 (3.14,5.72)	3.84 (2.99,5.24)	3.71 (3.09,5.68)	3.53 (2.77, 4.78)
PCV	Reference	9.50	14.50	20
Model comparison				
Log likelihood	− 768.00363	− 722.28902	− 754.00843	− 717.48826
Deviance	153,600.726	144,457.804	150,801.686	143,497.652

### Factors associated with untreated diarrhea

The model with smaller deviance and the largest likelihood (Model III) was best fit the data and the interpretation of the fixed effects was based on this model. Model III was adjusted for both individual and community-level factors. Age of child, family number, household wealth and community-level educational status were significantly associated with untreated diarrhea among children in Ethiopia. Children aged 6–11, 12–23, and 24–35 were 62% (AOR 0.38, 95% CI 0.19–0.79), 71% (AOR 0.29, 95% CI 0.15–0.60), and 51% (AOR 0.49, 95% CI 0.24–0.99) less likely not to received treatment for diarrhea as compared with a child aged 0–6 months respectively. Children from family number six and above had 64% (AOR 1.64, 95% CI 1.10–2.43) significantly contributing for untreated diarrhea than children from a family number less than or equal to five. Children from middle-wealth of family were 89% (AOR 1.89, 95% CI 1.17–3.30) more likely not to received treatment for diarrhea than those of children from richest wealth of family. Children from a community with high level of uneducated status had significantly contributed (AOR 2.78, 95% CI 1.07–3.44) for untreated diarrhea than children from a community with low level of uneducated status (Table 4).

**Table 4:** Factors associated with untreated diarrhea among under five children in the 2016 EDHS.

Variables	Categories	Diarrhea treatment		Odds ratio			
		Yes	No	COR	AOR1 (95% CI)	AOR2 (95% CI)	AOR3 (95% CI)
Age of child	0–6 m	28	63	1	1		1
	6–11 m	114	127	0.41 (0.21, 0.81)	0.40 (0.19, 0.81)***		0.38 (0.19, 0.79)***
	12–23	181	176	0.31 (0.16, 0.60)	0.30 (0.15, 0.60)***		0.30 (0.15, 0.60)***
	24–35 m	102	148	0.48 (0.25, 0.96)	0.50 (0.25, 1.02)		0.49 (0.24, 0.99)***
	> 35	98	190	0.81 (0.41, 1.60)	0.74 (0.37, 1.51)		0.73 (0.36, 1.49)
Respondent occupation	Not working	262	386	1.32 (0.961, 1.799)	1.40 (0.99, 1.98)		1.41 (0.99, 1.987)
	Working	261	318	1	1		1
Under-five children number	2 or less	473	602	1	1		1
	3 and above	51	101	1.59 (0.98, 2.57)	1.30 (0.77, 2.19)		1.28 (0.76, 1.99)
Family number	≤ 5	301	309	1	1		1
	6 and above	223	394	1.89 (1.37, 2.61)	1.67 (1.13, 2.45)***		1.64 (1.10, 2.43)***
Education	No formal education	278	489	2.13 (1.53, 2.96)	1.79 (1.20, 2.66)***		1.45 (0.93, 2.24)
	Formal education	246	214	1	1		1
Media	Yes	214	212	1	1		1
	No	310	491	1.59 (1.14, 2.21)	1.19 (0.81, 1.75)		1.18 (0.78, 1.80)
Wealth index	Poor	200	338	1.92 (1.32, 2.79)	1.53 (0.99, 2.37)		1.31 (0.81, 2.20)
	Middle	103	164	2.05 (1.34, 3.15)	2.05 (1.29, 3.26)***		1.89 (1.17, 3.04)***
	Rich	221	201	1	1		1
Age of women	15–24	150	157	1	1		1
	25–34	274	397	1.67 (1.16, 2.40)	1.28 (0.84, 1.94)		1.35 (0.88, 2.07)
	35–49	100	149	1.55 (0.97, 2.40)	0.84 (0.48, 1.47)		0.91 (0.52, 1.60)
Religion	Catholic	4	6	1	1		1
	Orthodox	203	248	0.20 (0.35, 1.10)	0.17 (0.03, 0.99)*		0.18 (0.03, 1.02)
	Protestant	120	193	0.44 (0.08, 2.38)	0.34 (0.06, 1.89)		0.38 (0.07, 2.08)
	Muslim	183	246	0.21 (0.04, 1.12)	0.15 (0.26, 0.82)*		0.19 (0.04, 1.03)
	Tradition	14	10	0.12 (0.15, 0.91)	0.04 (0.00, 0.34)*		0.13 (0.02, 1.01)
Community level poverty	Higher	228	375	1.86 (1.16, 2.98)		1.30 (0.77, 2.21)	1.52 (0.80, 2.89)
	Lower	296	328	1		1	1
Community level education	Higher	204	399	2.82 (1.77, 4.50)		2.44 (1.47, 4.05)***	1.97 (1.07, 3.64)**
	Lower	320	304	1		1	1
Region	metropolitan	15	11	1		1	1
	Small peripheral	28	26	1.82 (0.40, 4.81)		0.56 (0.15, 2.15)	0.54 (0.13, 2.27)
	Large central	481	666	2.20 (0.76, 6.39)		1.12 (0.35, 3.63)	1.14 (0.33, 2.23)
Residence	Urban	74	52	1		1	1
	Rural	450	651	2.81 (1.42, 5.54)		1.48 (0.66, 3.35)	0.90 (0.36, 2.23)
Community level media	Lower	258	416	1.84 (1.15, 2.95)		1.08 (0.64, 1.84)	0.90 (0.48, 1.68)
	Higher	266	287	1		1	1
Distance	Not big problem	235	277	1		1	1
	Big problem	288	427	1.34 (0.95, 1.87)		1.16 (0.82, 1.64)	1.04 (0.79, 1.51)

Significant values are in bold.

## Discussion

Diarrhea remains a high burden disease despite the availability of simple, affordable, and effective treatments. Understanding the impact of the socio-economic context on diarrheal illness is important, because it is not only the individual’s status, but the community characteristics that may also influence the risk of not to receive treatment for diarrhea infections.

In this study, the proportion of untreated diarrhea was 57.32% (95% CI 54.52–60.06%) of children in recent 2016 EDHS. This is an alarming sign that indicates the problem is still a huge concern which requires due attention to be paid by designing comprehensive health strategies. This finding is similar to a systematic review done in western Africa (57.6%) and eastern Africa (58.5%)^33^. However, lower than systemic review done in central Africa (67.4%) and other studies done in Ethiopia (73.5%) and (65%)^24,33,34^. Besides higher than study done in a Meklie Ethiopia (27.5%) and systematic review done in South Africa (41.1%)^33,35^. The reason for not treating diarrhea could be the perception of mothers/care givers that the episodes were not serious enough^36^. The other cause could be due to poor people in Ethiopia being especially disadvantaged in accessing quality healthcare because of their marginalized position in society^24^.

Children from family number six and above had one and half times not to receive treatment for diarrhea than children from a family number less than or equal to five. When the family size increased the chance of gating treatment for diarrhea is decreased. This study is also supported by other study conducted in Ethiopia^34^. Studies showed that mothers/caregivers high workload due to large family size brings about giving lesser attention to the sick child. Furthermore, financial restraints of large family members to visiting health facilities were related to family size^34,37^.

Untreated diarrhea among Children aged 6–11, 12–23, and 24–35 were 62%, 71% and 51% less likely not to received treatment as compared with a child aged 0–6 months. There have not been any further studies explaining this issue; this result indicates the relationship between treatment of diarrhea by age which implies that treatment of diarrhea increased as the age increased. This may be due to caregivers are more likely to seek facility care for children above one year with diarrhea; They believe that because the intestinal lumen of young children is not strong, diarrhea is natural and will go away on its own, however as the child's age increases, they consider diarrhea to be a disease and will seek treatment or facility care^38^.

Children from a middle wealth of family was near to 2 times more likely not to received treatment than those of children from a richest wealth of family. This study showed that the children from families with higher economic positions were more likely to take treatment for diarrhea rather than children from family’s middle economic positions. This finding is concurrent with studies conducted in Myanmar, Egypt, and other study done in Ethiopia^22,24,39^. It might be related to improvement of wealth has a significant effect on health-seeking behavior for childhood illness and lack of financial resources can create barriers to access healthcare services.

Children from a community with a high level of uneducated had approximately 2 times more likely not to received treatment than children from a community with a low level of uneducated. This study is also supported by studies conducted in India, Myanmar, Uganda, and Ethiopia^23,24,39,40^. This might be due to the fact that community education level can be associated with awareness of childhood illness and symptoms as well as the ability to timely interact with health care services^41,42^. Thus, investment in education may result in better access to health information for appropriate health-seeking behaviors.

### Strength and limitation of the study

The analysis was based on self-reported diarrhea treatment has reported by the caregiver which could be subject to recall bias. In spite of these limitations, the strength of our study lies in the unique characteristics of the DHS. The DHS is nationally representative, and allows for findings to be generalized across the entire country. Moreover, in this study, we were able to see the effect of socio-economic and other demographic predictors on the treatment of diarrhea among under-five children.

## Conclusion

The prevalence of untreated diarrhea among under-five children in Ethiopia is high. The study found that both the individual and community-level factors were associated with the outcome variable. Age of child, family number, household wealth, and community-level educational status were significantly associated with untreated diarrhea among under-five children in Ethiopia. It’s essential in targeting resources appropriately to raise the coverage of diarrhea treatment in the poor and the most vulnerable groups, particularly under-five children. The education status of the community should be increased and boosting the economic status of the family is essential.

## Data Availability

Data for this study were sourced from Demographic and Health surveys (DHS) and available at: https://www.dhsprogram.com/Data using the detail in the methods section of the document.

## Abbreviations

AOR: Adjusted odds ratio
CI: Confidence intervals
COR: Crude odds ratio
CSA: Central statistical agency
DHS: Demographic and health survey
EDHS: Ethiopian demographic and health survey
EA: Enumeration area
FMOH: Federal ministry of health
ICC: Intra-cluster correlation
ICCM: Integrated community case management
MOR: Median odds ratio
ORS: Oral rehydration salt
PCV: Proportional change in variance
SDGs: Sustainable development goals

## References

[1] Webair HH, Bin-Gouth AS (2013). Factors affecting health seeking behavior for common childhood illnesses in Yemen. Patient Prefer. Adher..

[2] UNICEF.org. Child survival and undefive mortality 2020.

[3] Guidline, W. Fact sheets of diarrhal disease. 2017.

[4] Lawn J, Blencowe H, Oza S (2014). The UN inter-agency group for child mortality estimation: Levels & trends in child mortality: Report 2014. New York: UNICEF: Every newborn: Progress, priorities, and potential beyond survival. Lancet.

[5] Demographic CSACEaIE. aHS. Key Indicators Report. Addis Ababa, Ethiopia, and Rockville, Maryland, USA. CSA and ICF 2016 (2016).

[6] Alebel A, Tesema C, Temesgen B (2018). Prevalence and determinants of diarrhea among under-five children in Ethiopia: A systematic review and meta-analysis. PLoS ONE.

[7] WHO. Diarrhoeal Disease: Fact Sheet. 2017 (accessed 15 Nov 2018).

[8] Beaugerie L, Sokol H (2012). Acute infectious diarrhea in adults: Epidemiology and management. Presse Med..

[9] Black, R. E. Progress in the use of ORS and zinc for the treatment of childhood diarrhea. *J. Glob. Health*. **9**(1) (2019). 10.7189/09.010101 PMC6344068 30701067

[10] Lewnard JA, Rogawski McQuade ET, Platts-Mills JA (2020). Incidence and etiology of clinically-attended, antibiotic-treated diarrhea among children under five years of age in low-and middle-income countries: Evidence from the Global Enteric Multicenter Study. PLoS Negl. Trop. Dis..

[11] Braimoh T, Danat I, Abubakar M (2021). Private health care market shaping and changes in inequities in childhood diarrhoea treatment coverage: Evidence from the analysis of baseline and endline surveys of an ORS and zinc scale-up program in Nigeria. Int. J. Equity Health..

[12] Masangwi S, Ferguson N, Grimason A (2016). Care-seeking for diarrhoea in Southern Malawi: Attitudes, practices and implications for diarrhoea control. Int. J. Environ. Res. Public Health.

[13] WHO/UNICEF. Diarrhea: Why children are still dying and what can be done accessed date (2022).

[14] UAO. 2019. Child health diarrheal disease (2018).

[15] UNICEF. Global databases based on Multiple Indicators Cluster Surveys. Demographic and Health Surveys and other nationally representative sources (2020).

[16] Ebrahim NB, Atteraya MS (2021). Oral rehydration salts therapy use among children under five years of age with diarrhea in Ethiopia. J. Public Health Res..

[17] Okoh BA, Alex-Hart BA (2014). Home management of diarrhoea by caregivers presenting at the diarrhoea training unit of a tertiary hospital in Southern Nigeria. Br. J. Med. Med. Res..

[18] Akombi BJ, Agho KE, Hall JJ (2017). Stunting and severe stunting among children under-5 years in Nigeria: A multilevel analysis. BMC Pediatr..

[19] Herba CM, Glover V, Ramchandani PG (2016). Maternal depression and mental health in early childhood: An examination of underlying mechanisms in low-income and middle-income countries. Lancet Psychiatry.

[20] Lépine J, Briley M (2011). The increasing burden of depression. Neuropsychiatr. Dis. Treat..

[21] Abuzerr S, Nasseri S, Yunesian M (2019). Prevalence of diarrheal illness and healthcare-seeking behavior by age-group and sex among the population of Gaza strip: A community-based cross-sectional study. BMC Public Health.

[22] Benova L, Campbell OM, Ploubidis GB (2015). Socio-economic inequalities in curative health-seeking for children in Egypt: Analysis of the 2008 Demographic and Health Survey. BMC Health Serv. Res..

[23] Bbaale E (2011). Determinants of diarrhoea and acute respiratory infection among under-fives in Uganda. Aust. Med. J..

[24] Ayalneh AA, Fetene DM, Lee TJ (2017). Inequalities in health care utilization for common childhood illnesses in Ethiopia: Evidence from the 2011 Ethiopian Demographic and Health Survey. Int. J. Equity Health..

[25] Aremu O, Lawoko S, Moradi T (2011). Socio-economic determinants in selecting childhood diarrhoea treatment options in Sub-Saharan Africa: A multilevel model. Ital. J. Pediatr..

[26] Assembly GJSTOW. Sustainable development goals. 2030 (2015).

[27] Bilal, N. K., Herbst, C. H., Zhao, F. *et al*. Health extension workers in Ethiopia: improved access and coverage for the rural poor. 433–443 (2011).

[28] Sarker AR, Sultana M, Mahumud RA (2016). Prevalence and health care-seeking behavior for childhood diarrheal disease in Bangladesh. Glob. Pediatr. Health..

[29] Adane M, Mengistie B, Mulat W (2017). Utilization of health facilities and predictors of health-seeking behavior for under-five children with acute diarrhea in slums of Addis Ababa, Ethiopia: A community-based cross-sectional study. J. Health Popul. Nutr..

[30] Aftab W, Shipton L, Rabbani F (2018). Exploring health care seeking knowledge, perceptions and practices for childhood diarrhea and pneumonia and their context in a rural Pakistani community. BMC Health Serv. Res..

[31] StataCrop SST (2015). Stata Statistical Software: Release 14.

[32] Mwangome FK, Holding PA, Songola KM (2012). Barriers to hospital delivery in a rural setting in Coast Province, Kenya: Community attitude and behaviours. Rural Remote Health..

[33] Akinyemi JO, Banda P, De Wet N (2019). Household relationships and healthcare seeking behaviour for common childhood illnesses in sub-Saharan Africa: A cross-national mixed effects analysis. BMC Health Serv. Res..

[34] Abegaz NT, Berhe H, Gebretekle GB (2019). Mothers/caregivers healthcare seeking behavior towards childhood illness in selected health centers in Addis Ababa, Ethiopia: A facility-based cross-sectional study. BMC Pediatr..

[35] Fissehaye T, Damte A, Fantahun A (2018). Health care seeking behaviour of mothers towards diarrheal disease of children less than 5 years in Mekelle city, North Ethiopia. BMC Res. Notes..

[36] Das SK, Nasrin D, Ahmed S (2013). Health care-seeking behavior for childhood diarrhea in Mirzapur, rural Bangladesh. Am. J. Trop. Med. Hyg..

[37] Gebretsadik, A., Worku, A., Berhane, Y. Less than one-third of caretakers sought formal health care facilities for common childhood illnesses in Ethiopia: Evidence from the 2011 Ethiopian demographic health survey. *Int. J. Fam. Med*. **2015** (2015). 10.1155/2015/516532 PMC4529949 26273479

[38] Kanté AM, Gutierrez HR, Larsen AM (2015). Childhood illness prevalence and health seeking behavior patterns in rural Tanzania. BMC Public Health.

[39] Lwin KS, Nomura S, Yoneoka D (2020). Associations between parental socioeconomic position and health-seeking behaviour for diarrhoea and acute respiratory infection among under-5 children in Myanmar: a cross-sectional study. BMJ Open.

[40] Sreeramareddy CT, Sathyanarayana T, Kumar HHJPO (2012). Utilization of health care services for childhood morbidity and associated factors in India: A national cross-sectional household survey. PLoS ONE.

[41] Noordam AC, Carvajal-Velez L, Sharkey AB (2015). Care seeking behaviour for children with suspected pneumonia in countries in sub-Saharan Africa with high pneumonia mortality. Soc. Sci. Med..

[42] Basu AM, Stephenson R (2005). Low levels of maternal education and the proximate determinants of childhood mortality: A little learning is not a dangerous thing. Soc. Sci. Med..

